# Modeling the spatial and temporal dynamics of foraging movements of humpback whales (*Megaptera novaeangliae*) in the Western Antarctic Peninsula

**DOI:** 10.1186/s40462-015-0041-x

**Published:** 2015-06-01

**Authors:** Corrie Curtice, David W Johnston, Hugh Ducklow, Nick Gales, Patrick N Halpin, Ari S Friedlaender

**Affiliations:** Marine Geospatial Ecology Lab, Nicholas School of the Environment, Duke University Marine Laboratory, Beaufort, NC 28516 USA; Division of Marine Science and Conservation, Nicholas School of the Environment, Duke University Marine Laboratory, Beaufort, NC 28516 USA; Lamont Doherty Earth Observatory, Columbia University, Palisades, NY 10964 USA; Australian Antarctic Division, Kingston, TAS Australia; Marine Geospatial Ecology Lab, Nicholas School of the Environment, Duke University, Durham, NC 27708 USA; Marine Mammal Institute, Hatfield Marine Science Center, Oregon State University, Newport, OR 97365 USA

**Keywords:** Humpback whale, Foraging, Western Antarctic Peninsula, Antarctic krill, Satellite telemetry, Space-time utilization distribution, Product kernel

## Abstract

**Background:**

A population of humpback whales (*Megaptera novaeangliae*) spends the austral summer feeding on Antarctic krill (*Euphausia superba)* along the Western Antarctic Peninsula (WAP). These whales acquire their annual energetic needs during an episodic feeding season in high latitude waters that must sustain long-distance migration and fasting on low-latitude breeding grounds. Antarctic krill are broadly distributed along the continental shelf and nearshore waters during the spring and early summer, and move closer to land during late summer and fall, where they overwinter under the protective and nutritional cover of sea ice. We apply a novel space-time utilization distribution method to test the hypothesis that humpback whale distribution reflects that of krill: spread broadly during summer with increasing proximity to shore and associated embayments during fall.

**Results:**

Humpback whales instrumented with satellite-linked positional telemetry tags (n = 5), show decreased home range size, amount of area used, and increased proximity to shore over the foraging season.

**Conclusions:**

This study applies a new method to model the movements of humpback whales in the WAP region throughout the feeding season, and presents a baseline for future observations of the seasonal changes in the movement patterns and foraging behavior of humpback whales (one of several krill-predators affected by climate-driven changes) in the WAP marine ecosystem. As the WAP continues to warm, it is prudent to understand the ecological relationships between sea-ice dependent krill and krill predators, as well as the interactions among recovering populations of krill predators that may be forced into competition for a shared food resource.

## Background

Migratory animals typically spend a portion of their annual life cycle in resource-rich feeding grounds [1]. While in these areas, animals typically acquire enough energy to fuel migrations to spatially and temporally disparate breeding and calving grounds that are sometimes resource limited. For larger marine mammals such as humpback whales (*Megaptera novaeangliae*), breeding and feeding grounds are often several thousands of kilometers apart and require vast amounts of energy and time to transit, highlighting the need to feed efficiently during the time they spend on the foraging grounds.

In marine ecosystems, resources are often patchy in both space and time. In the continental shelf waters along the Western side of the Antarctic Peninsula (WAP), nutrient rich circumpolar deep water from the Antarctic Circumpolar current intrudes into coastal areas via a series of deep canyons on the continental shelf [2,3]. This water mixes with phytoplankton-rich and less dense surface waters during summer [4], and a resulting lens of nutrient-rich and phytoplankton-laden water is entrained near the surface. Sunlight stimulates algal productivity in these waters, which is subsequently consumed by a myriad of lower and, eventually, upper trophic level predators [4].

Humpback whales are the most numerous baleen whale found in the nearshore waters along the WAP [5-8]. These whales breed in tropical waters near the equator in winter and feed during summer months in the high-latitude Antarctic waters [9]. Because of their large body size (adults reach up to 15 meters long and 40 tons in weight), they have extremely high energetic demands. These needs are met through an anatomically evolved bulk-filter feeding mechanism that allows them to process a volume of prey-laden water nearly equal to their body mass in a single feeding lunge [10]. Antarctic krill (*Euphausia superba*) are the dominant macro-zooplankton in WAP waters and are the primary component of humpback whale diets in this area [11]. Previous work has shown that the distribution and abundance of humpback whales around the WAP are best predicted by that of Antarctic krill [12]. As mobile predators with high energetic demands, it stands to reason that humpback whales will seek out areas with increased prey abundance, changing their distribution to reflect such prey changes throughout the feeding season.

During summer months Antarctic krill are abundant both at the marginal ice edge zone as winter sea ice retreats and throughout open continental shelf waters [13]. A portion of the adult population of krill can also be found offshore, where they deposit their eggs in deep water [13]. Thus, during summer months, krill are distributed broadly from nearshore to beyond the continental shelf. In autumn, krill appear to move inshore and towards sheltered bays where they coalesce into large aggregations that will be covered by sea ice formation [13], minimizing predation risk from diving predators including baleen whales [7,14]. It is also believed that the under-ice habitat offers ample food to feed juvenile krill over the winter [13]. Sea ice formation varies both latitudinally along the WAP and annually, especially along the western side of the Antarctic Peninsula, generally reaching its greatest extent between July-September [15].

**Figure 1:**
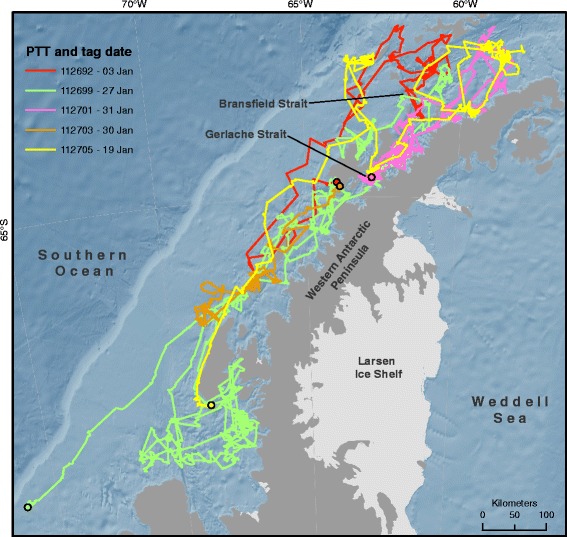
Whale movement tracks. Approximate paths of individual whale movement for five humpback whales tagged along the Western Antarctic Peninsula in 2012, with points showing the date and approximate location of Platform Transmitting Terminal (PTT) deployment.

Previous research on humpback whales in the Gerlache and Bransfield Strait areas of the WAP (see Figure 1 for these locations) found that whales exhibited both short and long-distance movements with relatively short residency times and variable-sized home ranges between presumed foraging areas during summer [16]. Recently, exceptional aggregations of both Antarctic krill [17] and humpback whales have been observed in nearshore bays late in the feeding season [7], with higher densities of whales than previously reported in these locations [8]. Given the known distribution of whales in summer months and the ultimate disposition of both whales and krill later in the feeding season, we hypothesize that the movement patterns and home ranges of humpback whales will reflect seasonal changes in the distribution and behavior of Antarctic krill. Specifically we predict that the area of whale movements will decrease over time, and that the overall distribution of humpback whales will become more proximate to shore over our study period, from the beginning of summer (January) to the end of the feeding season (June).

To test these predictions, we examined the spatial dispersion and coastal proximity of time-variant home ranges derived from satellite locations of Antarctic humpback whales in the WAP. Probabilistic home ranges are commonly used to describe an animal’s use of space [18], giving a probability of occurrence for an area. Van Winkle [19] described these as utilization distributions (UD) derived from two-dimensional animal locations, and Worton [20] describes the kernel estimator [21] as a robust, non-parametric, probability density function for determining the UD. Keating and Cherry [22] have expanded Van Winkle’s UD definition to include four dimensions, adding time and elevation (or depth in the case of marine animals), and expanded the traditional kernel method into a new “product kernel algorithm”. Here we applied this new product kernel method to a new taxonomic group (baleen whales) in a novel region (Antarctic Peninsula) and derived measures of space use to better understand the temporal movement patterns and behavior of these mobile ocean predators in a dynamic environment.

## Results

**Figure 2:**
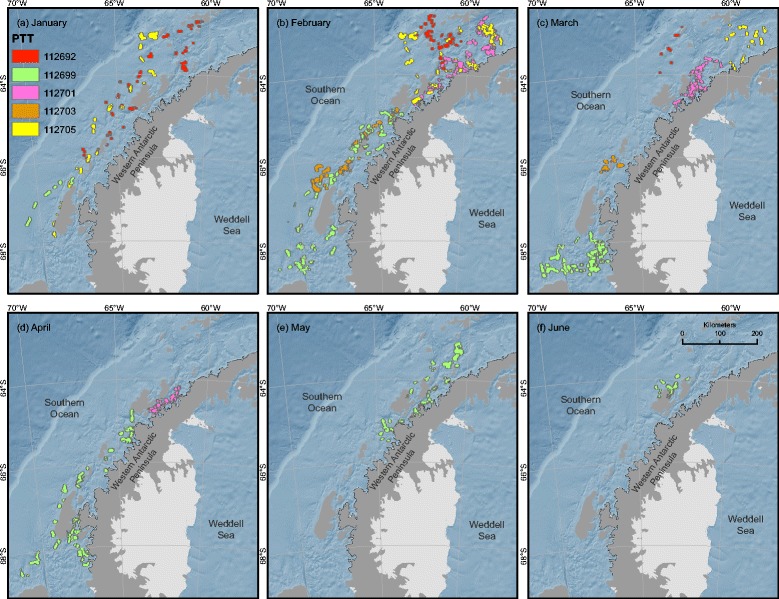
Home ranges over time for five humpback whales instrumented with Platform Transmitting Terminals (PTTs). The PTTs were deployed during January 2012, and recorded locations for varying durations (112692 – 65 days; 112699 – 140 days; 112701 – 81 days; 112703 – 38 days; 112705 – 52 days). Home ranges were calculated every 5^th^ day, for the period covering all five PTTs (3 January 2012 through 14 June 2012) as the 95^th^ percentile of the product kernel utilization distribution (UD). Home ranges were larger, more spread out, and further from the mainland in January **(a)** and February **(b)**, then begin to decrease in total area, spread, and distance to mainland during March through June (panels **c** - **f**). A black line shows the shoreline used to calculate distance from mainland for the centroid of each UD.

Five satellite-linked tags (Platform Transmitting Terminals [PTTs]) were deployed and remained active for between 38 and 140 days (Table 1; Figure 1). All of the PTTs were deployed during January 2012, with a difference of 28 days between the first and last deployments. Three PTTs (112692, 112703, 112705) stopped transmitting in early March (8 March, 8 March and 10 March respectively), and have the shortest durations (65, 38, and 52 days respectively) and are therefore skewed towards the beginning of the summer. The two remaining PTTs (112699, 112701) were the longest duration (140 and 81 days respectively), covering a later and longer period of the feeding season. Track lengths ranged from 1570 km to 9040 km (Table 1), providing a general measure of how much each whale moved. The quality of locations (Argos’ “Location Class”) equal to or greater than class 0 (the set of classes which have estimated error ranges associated with them) comprised 60% of the total locations used in the analysis (Table 1). Home ranges, defined as the 95% Utilization Distribution (UD) calculated with a spatio-temporal kernel density algorithm, were calculated for each of the five whales at up to 75 specific time steps, depending on the duration of the PTT (Figure 2).

**Table 1:** **Details of Platform Transmitting Terminal (PTT) tags**

**PTT**	**Deploy location**	**Deploy date**	**Last xmit**	**Days active**	**Received locations**	**Good locations**	**Filtered locations**	**Track length (km)**
112692	64°48'22"S 63°53'42"W	3-Jan-12	8-Mar-12	65	421	47	339	2294
112705	67°49'41"S 68°46'1" W	19-Jan-12	10-Mar-12	52	848	537	784	3375
112699	68°50'56"S 76°15'0"W	27-Jan-12	14-Jun-12	140	2783	2044	2600	9040
112703	64°48'18"S 63°53'56"W	30-Jan-12	8-Mar-12	38	438	201	397	1570
112701	64°43'34"S 62°48'43"W	31-Jan-12	21-Apr-12	81	1099	358	983	3467
				**Total**	**5589**	**3187**	**5103**	

Details include the date the PTT was deployed on the whale, the date of the last transmission to the Argos network, duration for which the PTT was active, total number of transmissions received, number of received locations that were of a “good” location class (0, 1, 2, or 3 - the classes for which error radius information is available), the number of locations remaining after a speed, distance and angle filter was applied, and the total track length.

### Model results

All tested models used Day of Year or month with and without distance to mainland as potential fixed effects predictors, and all tested models used PTT as a random effect to account for individual whale variations in movement (see Table 2 for full model results). Each model also included the temporal fixed effect (Day of Year or month) as a random slope. When AIC values for models were within two AIC units of the model with the lowest AIC score, the most simplistic model of that set was chosen [23]. Models were also evaluated based on the p-value and chi-square results when compared via the ANOVA (analysis of variance) likelihood ratio test to a “null” model with only distance to mainland as the fixed effect.

**Table 2:** **Model results for home range area and centroid pairwise distance**

**Model**	**Fixed effects**	**Random effect**	**Random slope**	**Response**	**SE**	**AIC**	Δ**AIC**	***χ*** **2**	**DF**	***p***
**Home range area** **(km** ^**2**^ **)**	**Month**	**PTT**	**Month|PTT**	**−215.7**	**36.8**	**1092**	**2**	**7.68**	**1**	**0.006**
	Month	PTT	Month|PTT	−214.9	38.3	1091	1	11.28	2	0.004
	+ DTM			2.7	1.4					
	DoY	PTT	DoY|PTT	−5.8	1.3	1094	4	2.27	1	0.132
	DoY	PTT		−6.5	1.2					
	+ DTM		DoY|PTT	3.4	1.3	1090	0	8.08	2	0.018
**Centroid pairwise distance (km)**	**Month**	**PTT**	**Month|PTT**	**−21.3**	**4.7**	**719**	**2**	**5.39**	**1**	**0.020**
	Month	PTT		−21.454	5.451					
	+ DTM		Month|PTT	0.285	0.145	717	0	9.72	2	0.008
	DoY	PTT	DoY|PTT	−0.6	0.1352	722	5	5.00	1	0.025

P-value and *χ*2 values were obtained by analysis of variance tests of each of the full models against a null model. Null models had Distance To Mainland (DTM) as the only fixed effect, to determine if month or Day of Year (DoY) was a significant predictor variable. DoY + DTM centroid pairwise distance model failed to converge and has been omitted. The Platform Transmitting Terminal (PTT) is unique to each whale, and was used as a random effect with by-whale random slope to capture the variation in movements between individual whales. Response is the unit change in the dependent variable (home range area in km^2^ or centroid pairwise distance in km) per unit increase in the fixed effect variable(s). For example, for each unit increase in month, the home range area decreased by 215.7 km^2^ with a standard error of ±36.8 km^2^. The selected model is bolded. The most parsimonious model within two Akaike information criterion (AIC) units of the lowest AIC was selected. SE = Standard Error; DF = Degrees of Freedom; p = p-value.

Three of the four home range area models showed significant difference from the null models and were within two AIC units of the model with the lowest AIC; the selected model with only month as a predictor (*χ*^2^(1) = 7.68, *p* = 0.006) was the most parsimonious of the three. With each increase in month, there was a corresponding decrease in home range area of 215.7 km^2^ ± 36.8 km^2^ (standard errors). The models with distance to mainland as predictors also show that as distance to mainland increased, the home range area also increased, indicating that when further from the mainland the whales range over a larger area.

The distances between the centroids of each home range area for each whale is a measure for the range or spread of each whale over the course of the foraging season [24]. If the whales move closer to shore, presumably following the movement of krill, the distances between the 95% UD polygons (representing the general “home range” for the 5-day spread around a given date) should decrease over time. Three models showed significant differences from the null models (one model failed to converge); the selected model with only month as the predictor (*χ*^2^(1) = 5.39, *p* = 0.02) was more parsimonious than the lowest AIC scoring model. For each increase in month the model shows a corresponding decrease in the pairwise distance of centroids of 21.3 km ± 4.7 km (standard errors).

## Discussion

The results of our spatial analyses indicate that the distribution and movement patterns of satellite-tagged humpback whales on Antarctic foraging grounds change significantly over the course of the feeding season (approximately January to June). Both of our spatial metrics - time-variant home range area and pairwise home range centroid distance - decrease as a function of time and proximity to shore. We believe that these results are additional evidence that humpback whales move in concert with seasonal changes in the broad-scale distribution of their main prey, Antarctic krill [13]. During January, the time of our PTT deployments, krill are generally dispersed across the nearshore and continental shelf waters of the WAP [14]. Over the course of the summer months, krill have been shown to move inshore and aggregate into denser patches [7,14]. Based on these previous studies of krill movement, our results provide supporting evidence that whales track the movement of their primary prey through the summer months, adjusting their movements to maintain proximity to this important resource, resulting in increased whale density in the nearshore regions of the WAP [8].

In the WAP region, a suite of predators relies on krill as a primary food item. In addition to baleen whales (including humpback, minke whales - *Balaenoptera bonaerensis*, and fin whales - *Balaenoptera physalus*), several penguin, seabird and seal species acquire the vast majority of their energy from Antarctic krill [25]. While these animals share a common prey, they exhibit markedly differently life history strategies that affect their foraging patterns and movement ecology. For example, during summer months, Adelie penguins (*Pygoscelis adeliae*) and gentoo penguins (*Pygoscelis papua*) are considered central-place foragers that come and go from terrestrial nesting sites frequently to provision and rear growing chicks. This requires the penguins to stay in close spatial proximity (15–60 km [26,27]) to such areas, and the breeding success of penguins at a particular breeding rookery depends largely on local krill abundance [28]. Crabaeater (*Lobodon carcinophagus*), leopard (*Hydrurga leptonyx*), and Antarctic fur seals (*Arctocephalus gazella*) also rely on krill as a food item in this region [29]. While they are not necessarily dependent on returning to rookeries to provision pups, they are limited in their foraging ranges by the presence of suitable haul-out areas where they can rest and avoid predators. These typically take the form of sea ice floes or rocky coastlines. Therefore, like penguins, krill-dependent seals are limited in their foraging ranges by physical substrate rather than directly by the distribution of their prey [12].

In contrast, humpback whale distribution is best predicted by the distribution of their prey [6], and these whales are not bound by the constraints of central place foragers [12]. Humpback whales spend summer months on foraging grounds replenishing lost energy and adding additional energy stores to fuel long distance migrations to tropical calving/breeding grounds [9]. Because they typically do not feed during migrations or on their breeding/calving grounds, humpback whales must acquire enough energy during summer months to support their energetic demands for the entire year [30]. It is therefore important for them to maximize their time on feeding grounds and maintain proximity to the highest densities of prey available to them. Our results support previous work that was based on visual sightings of whales over short periods of time [7,8,12,31], and potentially increases our understanding of the spatial relationship between humpback whales and krill over longer time periods. Linking work of long duration satellite tags with long duration observations of krill distribution [31], sea ice [15] and oceanographic conditions [32] will deepen this understanding.

While krill can be found in the nearshore waters around the WAP during summer months, they are also more specifically distributed in relation to several physical features that likely enhance local productivity. Krill are known to feed and aggregate at the marginal sea ice edge as ice retreats [13], and it has also been hypothesized that krill aggregate proximate to deep water canyons that allow nutrient-rich Circumpolar Deep water to move inshore and be upwelled, creating ideal conditions for primary producers and consumers [3]. This notion that, over time, these deep canyons provide predictable food resources for krill predators has been the focus of several long-term studies on the location of penguin rookeries around the WAP [28,33]. As the long photo-period of summer months begins to wane, it is believed that Antarctic krill begin to move inshore and into areas where they will overwinter [14]. Several studies have documented this offshore-inshore migration of krill into nearshore bays where krill eventually coalesce into massive aggregations [7,14]. It is theorized that krill make these inshore migrations to seek shelter under the cover of annual sea ice that limits access from air-breathing predators, and allows them to survive in large, dense aggregations until the following spring. Gerlache strait, a focal area in our study, is consistently the last part of the WAP to be covered with sea ice, typically beginning around June, giving whales longer foraging access to the aggregated krill [13].

Humpback whales are known to feed (via lunging) between 300–900 times in a 24-hour period, and must recover the energy used by feeding on high densities of krill [34]. It is likely then that humpback whales will graze local krill abundances below a level that is no longer energetically profitable and will move to a new location to feed when this level is reached [35]. During summer months it appears that krill are patchy and distributed in discrete aggregations across the southern WAP region and along the shelf break [36,37] and humpback whales would likely need to move frequently in search of suitable prey densities. The results of our analyses support this hypothesis, with whales having larger foraging ranges that are farther from the coastline earlier in the feeding season. The movement patterns of the tagged humpback whales in this study therefore may reflect the general pattern of seasonal krill movements, from a lower density offshore distribution into higher density near shore aggregations [7].

Our methods provide a metric to assess time-space use of a large and mobile marine predator. The new methods used in this study which show the progression of animal space use over time, could, in concert with concurrent prey studies lead to a new approach to evaluating the ecological relationship between predators and their prey (or other environmental features that provide context for behaviors) and may be applicable to a broad range of taxonomic groups in marine ecosystems.

### Caveats and considerations

Previously determined ecological relationships between humpback whales and krill in the WAP region provide for strong inference that the primary driver affecting the distribution and movement pattern of these whales is indeed that of Antarctic krill [6,12]. A competing hypothesis regarding the observed movement patterns of humpback whales is that the animals will, over time, graze down krill resources below a profitable threshold level (marginal value theory) and move from patch to patch in order to satisfy their energetic demands [35,38]. Currently, baleen whales are still at a fraction of their historic population levels and there is no evidence to suggest that krill are a limited resource in the area [39]. Thus, while whales are likely to graze patches at a very local level, their ability to diminish resources across a broad area is unlikely. If this were the case, whales would increase their search radius over time to find resources outside of where they have already grazed, something that is not supported by the data we have presented.

Our results only provide information for a single year, with a modest sample size, and there is likely to be inter-annual variability in environmental conditions in this region that may influence the magnitude of the relationship between krill distributions and the space use of humpback whales. However, there are no data to suggest that the previously determined relationships between the distribution of whales and krill will fundamentally change over such short time frames.

PTT deployments were generally of short duration, with only two of the five reporting data after early March. Since we are trying to capture changes in behavior throughout the course of the feeding season, which can last into June in this area of the WAP, it is possible that the three shorter deployments are not fully capturing the transition towards more constricted, near shore movements. Longer deployments would help address this consideration, and help support our theory that the constricting use of space over time applies broadly to the humpback whales foraging along the WAP.

Other environmental parameters also contribute to humpback whale distribution (e.g. sea surface temperature, deep temperature maximum, amount and extent of sea ice cover) however several analytic models all show krill as the most significant determinate [6,12]. Examination of the Passive Microwave Data from the National Snow & Ice Data Center for the months of this study (January – June 2012) show the Gerlache and Bransfield Straits areas to be ice free from February through sometime in June [40], supporting our theory that the whales are following their prey, and not altering their home ranges over time to avoid ice cover.

Another factor potentially influencing results is the battery-life of the PTTs, although it is unlikely that battery degradation and related transmission loss altered the outcome of our analysis. In this study, the tags were programmed to conserve the life of two lithium ion batteries on-board the tag by duty cycling on for four hours, then off for eight hours. A reduction in battery power over time may potentially alter the number of successful transmissions, however our data show only a slight degradation of transmission rate. It’s more likely that the tags stopped transmitting after catastrophic failures such as the tag falling off, salt water invasion in the tag, or antennae fouling or breaking. Other factors could also confound transmission rate, such as the behavior of the whale – if actively feeding the whale will spend less time at the surface, giving less opportunity for a location to be obtained [34].

## Conclusions

Despite the small sample size, this study provides initial results of how the movements of humpback whales in the WAP region are likely related to the seasonal change in distribution of their primary prey, Antarctic krill. Our application of a novel method for showing changes in space use over time present a baseline for future observations of the seasonal changes in the movement patterns and foraging behavior of humpback whales, and potentially other Antarctic krill predators. The amount and persistence of sea ice around the western side of the WAP has decreased significantly since 1979 [41] while air temperatures have risen [42]. The life history of Antarctic krill is intimately tied to sea ice cover and the documented changes that we are currently witnessing have been implicated in the decrease of krill in this region [43]. As conditions continue to change, it is prudent to understand the ecological relationships between krill and krill predators in the WAP as well as interactions among krill predators that may be increasingly subjected to competition for a shared food resource.

## Methods

During January 2012, Wildlife Computer (Redmond, WA, USA) SPOT5 Platform Transmitting Terminals (PTTs) were attached to six humpback whales in the continental shelf waters of the WAP (Figure 1) [44]. Each PTT is contained in a stainless steel custom housing that penetrates the whale’s skin and hypodermis up to 290 mm deep, and is anchored in the tissues beneath the blubber layer with stainless steel foldable barbs. PTTs were kept in sealed sterilized packages until deployment from a Mark V Zodiac rigid-hulled inflatable boat with a 40-hp 4-stroke engine using a compressed air gun set at a pressure between 7.5 and 10 bar. Whales were approached at idle speed and from a perpendicular or oblique rear angle to reduce disturbance, and the PTTs were deployed at a range of 3-8 m. PTTs were placed high on the dorsal surface of the whale, in the vicinity of the dorsal fin, to maximize antenna exposure each time the animal surfaced. The dorsal fins and flukes of each tagged animal were photographed for individual identification.

SPOT5 PTTs are satellite linked via the Argos System. All were programmed to transmit daily during the hours 00:00 to 04:00 and 12:00 to 16:00 (GMT) and were activated via the salt-water switch with the first dive after tagging. Locations obtained through Argos have varying levels of estimated error. Each location is coded with a location class (LC) starting with Z, B, and A which have no predicted estimated error, and LC 0, 1, 2, and 3 which have an associated 1-sigma error radius of approximately >1500 m, <1500 m, <500 m, and <250 m, respectively [45]. Locations with an LC of Z were not included in the analysis because they are considered invalid by Argos. Remaining locations were filtered using the sdafilter function in the argosfilter [46] package in the R development environment [47] to remove improbable locations based on swimming speed, distance between locations, and turning angle between locations. A maximum estimated swimming speed of 5 m/s was used. All locations were projected to UTM Zone 20 South. Location points for each PTT were transformed into track lines using the “Points to Line” tool [48], and the length calculated via the calculate geometry tool. One track that was only three days in duration was removed from the data set.

To evaluate changes in habitat use of tagged humpback whales over the feeding season, we used a robust product kernel method [22] as implemented in the adehabitat package for R [49]. This method extends the traditional utilization distribution (UD) method [21] by allowing four dimensions to be modeled (*x, y, z, t*), where *z* represents elevation/depth and *t* represents time in either linear or circular units. The tags used in this study did not record dive behavior, so the *z* dimension was not used in this analysis. Bandwidths were chosen for *x* and *y* as 5000 m, and for *t* as 5-days, each based on initial exploration of the data [21].

Using the full date range of location data for all five animals (2012 January 3 –2012 June 14, 162 days, Table 1), on every 5^th^ day we calculated the time- and space-smoothed UD for each whale whose track existed on that date. For each UD, the 95% isopleth was extracted as a polygon and used as the extent for the home range [20] for the 5-day spread around that date (Figure 2). In ArcGIS [48], land was erased from the polygons using an Antarctic land shapefile from the Antarctic Digital Database [50] and the area (km^2^) of each resulting polygon was calculated. The centroid of each land-adjusted polygon was then determined and used to calculate distance (km) to the WAP mainland (excluding islands), hereafter referred to as DTM. These values were then averaged for each whale for each time-smoothed UD around each date to get a single per-date DTM, to be used in the regression modeling. The area of the combined 95% UD polygons (the total summed home range) for each whale on each date was used for regression modeling. Additionally, we averaged the pairwise distances (PWD) of the centroids among multiple polygons for each date for each whale to get a quantitative measure of each respective UD’s spatial spread. These values represent the total range of a whale in a given 10-day period (the date of the UD smoothed by 5 days); our hypothesis indicates that these values should also decrease over the duration of the summer feeding season, as the whales spend increasing amounts of time in smaller areas feeding on krill aggregations.

We used R and lme4 [51] to perform linear mixed effects analyses to model humpback whale home range area and PWD. DTM, Day of Year and month were used as potential fixed effects, and the PTT was used as a random effect with by-whale random slopes. P-values were obtained by ANOVA (analysis of variance) likelihood ratio tests of each of the full models against a null model with DTM as the only fixed effect.
